# The Significance of SIX1 as a Prognostic Biomarker for Survival Outcome in Various Cancer Patients: A Systematic Review and Meta-Analysis

**DOI:** 10.3389/fonc.2021.622331

**Published:** 2021-10-21

**Authors:** Guang Zhu, Ying Liu, Lei Zhao, Zhenhua Lin, Yingshi Piao

**Affiliations:** ^1^ Guangdong Provincial Education Department Key Laboratory of Nano-Immunoregulation Tumour Microenvironment, The Second Affiliated Hospital, Guangzhou Medical University, Guangzhou, China; ^2^ Tumor Research Center, Medical School of Yanbian University, Key Laboratory of Pathobiology of High Frequency Oncology in Ethnic Minority Areas (Yanbian University), State Ethnic Affairs Commission, Yanji, China; ^3^ Key Laboratory of Science and Technology Department of Jilin Province, Key Laboratory of Changbai Mountain Natural Medicine of Ministry of Education, Yanbian University, Yanji, China

**Keywords:** Sine Oculis Homeobox Homolog 1, cancer, meta-analysis, prognosis, TCGA

## Abstract

Sine Oculis Homeobox Homolog 1 (SIX1) is reported to promote cancer initiation and progression in many preclinical models and is demonstrated in human cancer tissues. However, the correlation between SIX1 and cancer patients’ prognosis has not yet been systematically evaluated. Therefore, we performed a systematic review and meta-analysis in various human cancer types and extracted some data from TCGA datasets for further verification and perfection. We constructed 27 studies and estimated the association between SIX1 expression in various cancer patients’ overall survival and verified with TCGA datasets. Twenty-seven studies with 4899 patients are include in the analysis of overall, and disease-free survival, most of them were retrospective. The pooled hazard ratios (HRs) for overall and disease-free survival in high SIX1 expression patients were 1.54 (95% CI: 1.32-1.80, *P*<0.00001) and 1.83 (95% CI: 1.31-2.55, *P*=0.0004) respectively. On subgroup analysis classified in cancer type, high SIX1 expression was associated with poor overall survival in patients with hepatocellular carcinoma (HR 1.50; 95% CI: 1.17-1.93, *P* =0.001), breast cancer (HR 1.31; 95% CI: 1.10-1.55, *P* =0.002) and esophageal squamous cell carcinoma (HR 1.89; 95% CI: 1.42-2.52, *P*<0.0001). Next, we utilized TCGA online datasets, and the consistent results were verified in various cancer types. SIX1 expression indicated its potential to serve as a cancer biomarker and deliver prognostic information in various cancer patients. More works still need to improve the understandings of SIX1 expression and prognosis in different cancer types.

## Introduction

Cancer is now being predicted as a leading cause of deaths based on the latest global cancer statistic report, there are 24.5 million new cancer diagnoses and 9.6 million deaths each year (1). The features of most cancers are heterogenous, depends on the treatment response, recurrence, and the cancer metastasis potential. Biomarkers that annotated this different feature in different tumor types and stages, either independently or involved in the current tumor stages can offer deep understandings and help to guide the best clinical treatment, like radical surgery or chemoradiotherapy. This approach was approved by both follow-up data collections and the statistics obtained by cancer patients’ outcome. However, despite the advantages that cancer biomarkers offered, failures in transforming discoveries into clinical models or lacking prospective biomarker validation to guide clinical treatment properly still troubles cancer experts and clinical treatments.

In the past two decades, many studies demonstrated that sine oculis homeobox 1 (SIX1) serve as a regulator in organ generation and play essential roles both in tumorigenesis and progression (2). In vertebrates, the SIX gene family is characterized by the SIX-type homeodomain (HD, 60 amino acids) and SIX domain (SD, 110-115 amino acids). SIX family genes have been discovered in many kinds of species and is highly conservative. One family member, the SIX homeobox 1 (SIX1), is extensively investigated. SIX1 is reported involving in the development of tissues, like muscle (3), kidney (4), sensory organs (5), and auditory system (6). Recently, much attention was switched to the roles of SIX1 in tumorigenesis. Reports showed that SIX1 participates in various cancers initiation, including hepatocellular carcinoma (7), breast cancer (8), ovarian cancer (9), cervical cancer (10), osteosarcoma (11), colorectal cancer (12), and Wilms tumors (13). Meanwhile, SIX1 has also been verified in promoting cancer progression, accelerating cancer cell metabolism and progression.

Given that the complexity and its various roles play, SIX1 is essential in the initiation and progression of primary cancer to a distant metastasis and finally lead to an untreatable late stage (14, 15). However, determining the prognostic benefits of SIX1 expression in cancer patients is unclear. The prognostic role of total SIX family member protein expression had been estimated by two meta-analysis articles in 2016, both was estimated in single cancer type, one was estimated in breast cancer (8), and the other was in lung cancer (16), as new research appeared, we intend to conduct a new meta-analysis and to expound the potential prognostic value of specific SIX1 in depth. To address this issue, we utilized a systematic meta-analysis of SIX1 protein expression in tumor tissues with a variable being high *versus* low SIX1 expression level. We estimated the correlation between SIX1 expression level and prognosis in multiple cancers. The validation with the The Cancer Genome Atlas (TCGA) online datasets were added for further data perfection and supplementary of this study.

Our aims were to investigate whether SIX1 expression predicts survival outcomes in cancer patients, and whether SIX1 quantization can be considered as a helpful biomarker in various cancers.

## Materials and Methods

### Publication Search

This study followed the Preferred Reporting Items for Systematic Reviews and Meta-Analysis (PRISMA) (17). We utilized a systematic search based on the PubMed, Web of Science and Embase database from January 1, 2006, to August 31, 2020, using both keywords. Our search keywords were: (“cancer” OR “tumor” OR “carcinoma”) and (“SIX1” OR “Sine Oculis Homeobox Homolog 1”) and (“prognosis” OR “prognostic” OR “outcome”). References or associated research were reviewed and include as potential articles.

### Inclusion and Exclusion Criteria

The articles selection process was done by two doctors (Dr. Lin and Dr. Piao). The included criteria were as follows: (1) reported the correlation of the expression of SIX1 and the survival data of patients; (2) performed the relevant clinicopathological features. The exclusion articles were as follows: (1) studies not based on human; (2) unreachable/insufficient Hazard ratios (HRs); (3) case reports, reviews, or meta-analysis; (4) repetitive patients. Studies on human with solid cancer report and presented the effect of SIX1 on overall survival, and/or disease/recurrence-free survivals were included. Studies were excluded if they published based on duplicated data.

### Statistical Analysis

Data was cross-checked by (Dr. Lin and Dr. Piao) independently. In all included studies, the independent variable survey was the SIX1 expression defined as high or low. The natural logarithm and standard error of hazard ratio were gathered for the prognosis in each study. Pooled estimates were performed using forest plots and analyzed by using Review Manager Version 5.3. Heterogeneity was considered high, medium, or low if *I^2^* above 75%, 50-75% or below 50%, respectively (18). Digitizer 4.1 software was used to extract data from the Kaplan-Meier (K-M) plot, if the HRs and its 95% confidence inter (CIs) offered indirectly by the article. In addition, the included studies were all assessed by using the Newcastle-Ottawa Scale (NOS) (19).

Funnel plots were constructed for evaluating the overall and disease-free survival analyses. Subgroup analysis was aimed to perform the correlation between SIX1 expression and the prognosis in various cancers for overall survival. *P*-value<0.05 considered as statistically significant.

Our meta-analysis was based on the Stata 12.0 software (Stata Corporation, College Station, TX, United States) and Review Manager Version 5.3 (Cochrane Collaboration, Oxford, UK). The prognostic value of SIX1 on OS, DFS was performed by pooled HRs and 95% CIs. Besides, odds ratios (ORs) with corresponding 95% CIs were performed to analyze the association between SIX1 and clinicopathological features. Chi square-based Cochran Q test and *I^2^* test were calculated to determine the heterogeneity among these articles. *I^2^* > 50% or *P*-value < 0.05 was considered for being a significant heterogeneity and a random-effect model would be used, otherwise, a fixed model will be used. The effect of covariates was evaluated by regression analysis. The sensitivity and publication bias were also performed. *P* < 0.05 was considered statistically significant with two-sides.

### Analysis Using TCGA Online Database

Data of SIX1 expression and clinicopathological parameters in TCGA were obtained from the Gene Expression Profiling Interactive Analysis (GEPIA, http://gepia.cancer-pku.cn) (20) and the KM-plotter (http://kmplot.com/analysis/index.php?p=background). To generate the K-M survival analysis and the overall survival plots, SIX1 expression levels were classified into low/median or high. The difference between two groups was conducted by Log-rank test.

### Mechanism Prediction of SIX1 and Protein Interaction Network

We obtained the data from STRING database (http://string-db.org/) (21), an online common software, to find SIX1related genes and to provide an assessment for the protein-protein interactions (PPI) of SIX1 and its related genes.

## Results

### Search Results

**Figure 1:**
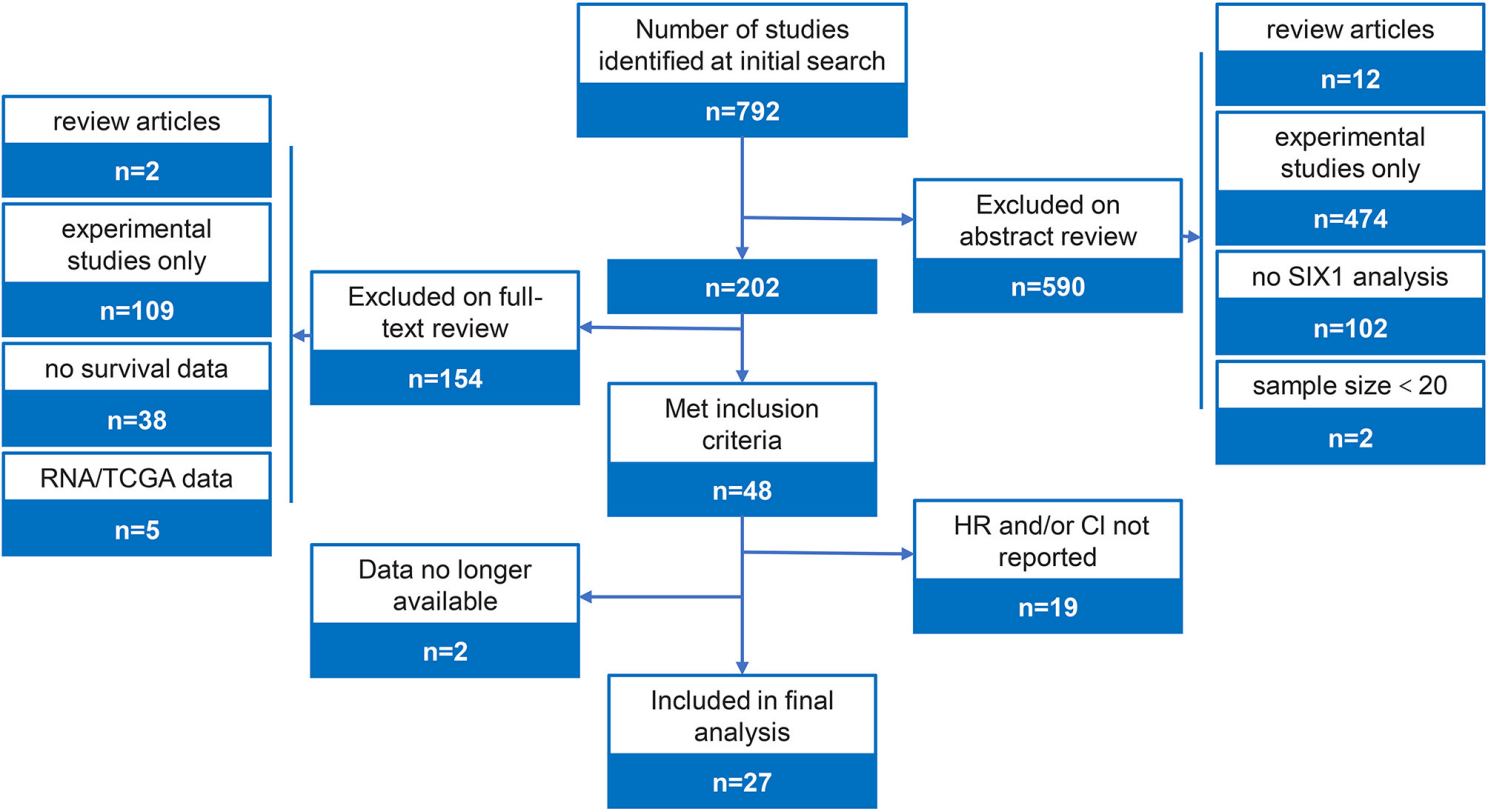
Study flow diagram and selection criteria.

The study flow figure is shown in **Figure 1**. A total of 27 studies were included in this meta-analysis with a total 4899 patients (**Table 1**). 27 studies provided overall survival data, 5 among which also reported disease-free survival data. Some other studies failed to report sufficient data and were excluded. 202 articles remained after scanning the titles and abstracts, and among the 202 studies, 109 were experimental studies only, 2 were excluded for review articles, 38 were excluded for no survival data reported, 19 were excluded for insufficient HRs or other data, 2 were excluded because the data is no longer available, and 5 analysis was based on TCGA data base. Finally, 27 eligible studies were including in this meta-analysis (22–48). These eligible researches contained 4899 patients, involved 12 types of cancers, including the hepatocellular carcinoma (n=3), colorectal cancer (n=2), breast cancer (n=10), esophageal squamous cell carcinoma (n=3), gastric cancer (n=3), pancreatic cancer (n=1), lung Adenocarcinoma (n=1), Osteosarcoma (n=1), Melanoma (n=1), Prostate cancer (n=1), and Glioma (n=1). In these studies, SIX1 expression levels were estimated by qPCR or immunohistochemistry (IHC). The characteristics of the articles were listed in **Table 1**.

**Table 1:** Characteristics of studies in this meta-analysis.

First author	Year	Country	No. of patient	Cancer Type	Method	Cut-off	Outcome	U/M	Analysis	NOS
Ng et al	2006	China	103	Hepatocellular Carcinoma	qPCR	≥median	OS	U/M	K-M curve	8
Kong et al	2014	China	284	Hepatocellular Carcinoma	IHC	≥26% positve cells	OS, DFS	U/M	K-M curve	9
Chen et al	2019	China	23	Hepatocellular Carcinoma	qPCR	≥median	OS, DFS	─	K-M curve	8
Ono et al	2012	Japan	120	Colorectal cancer	IHC	IHC score>1	OS	─	K-M curve	7
Kahlert et al	2015	Germany	945	Colorectal cancer	IHC	IHC score≥4	OS, DFS	U/M	K-M curve	7
Haidan et al	2014	China	262	Breast cancer	IHC	≥26% positve cells	OS	U/M	K-M curve	8
Hennessy et al	2009	USA	89	Breast cancer	qPCR	≥median	OS	─	Reported	9
Pawitan et al	2005	Sweden	159	Breast cancer	qPCR	─	OS	─	Reported	8
Bild et al	2006	USA	158	Breast cancer	qPCR	≥median	OS	─	Reported	9
Desmedt (1) et al	2007	Belgium	198	Breast cancer	qPCR	─	OS	─	Reported	9
Desmedt (2) et al	2011	Belgium	120	Breast cancer	qPCR	≥median	OS	─	Reported	8
Kao et al	2011	Taiwan	327	Breast cancer	qPCR	≥median	OS	─	Reported	9
Dedeurwaerder et al	2011	Belgium	88	Breast cancer	qPCR	─	OS	─	Reported	8
Heikkinen et al	2011	Finland	183	Breast cancer	qPCR	≥median	OS	─	Reported	8
Terunum et al	2014	USA	61	Breast cancer	qPCR	≥median	OS	─	Reported	9
Nishimura et al	2017	Japan	60	Esophageal squamous cell carcinoma	qPCR	≥median	OS	─	K-M curve	7
Wei et al	2013	China	292	Esophageal squamous cell carcinoma	IHC	IHC score≥4	OS	U	K-M curve	7
He et al	2017	China	119	Esophageal squamous cell carcinoma	IHC	IHC score≥4	OS	M	K-M curve	7
Du et al	2017	China	40	Gastric cancer	IHC	IHC score≥4	OS	─	K-M curve	7
Xie et al	2018	China	208	Gastric cancer	IHC	IHC score≥4	OS	U/M	K-M curve	7
Lv et al	2014	China	163	Gastric adenocarcinoma	IHC	≥26% positve cells	OS, DFS	U/M	K-M curve	8
Jin et al	2014	China	148	Pancreatic ductal cancer	IHC	≥26% positve cells	OS	U/M	K-M curve	8
Mimae et al	2011	Japan	64	Lung Adenocarcinoma	qPCR	≥median	OS	─	K-M curve	9
Chao et al	2017	China	100	Osteosarcoma	IHC	IHC score>1	OS, DFS	U/M	K-M curve	8
Monteiro et al	2019	Germany	278	Melanoma	qPCR	≥median	OS	M	K-M curve	9
Zeng et al	2015	China	144	Prostate cancer	IHC	IHC score≥4	OS	U/M	K-M curve	7
Zhang et al	2017	China	163	Glioma	IHC	IHC score≥4	OS	U/M	K-M curve	7

IHC, Immunohistochemistry; OS, Overall Survival; DFS, Disease-free Survival; NOS, Newcastle-Ottawa Scale; U/M, Univariate/Multivariate survival analysis; K-M curve, Kaplan-Meier survival curve; “—”, Not available/not reported.

### Study Demographics

The demographics of the included studies were listed in **Table 1**. There are 3 studies in hepatocellular carcinoma (410 patients), 2 studies in colorectal cancer (1065 patients), 10 studies in breast cancer (1645 patients), 3 studies in esophageal squamous carcinoma (471 patients), 3 studies in gastric cancer (411 patients). One each study for other six cancer types: pancreatic ductal cancer (148 patients), lung adenocarcinoma (64 patients), osteosarcoma (100 patients), melanoma (278 patients), prostate cancer (144 patients), glioma (163 patients). (**Tables 1**, **2**)

**Table 2:** Subgroup analysis of pooled HR for OS.

Categories	Number of studies	Number of patients	Pooled HR (95%CI)		Heterogeneity	
			Fix/Random	*p*-value	*I^2^* (%)	*P_H_*
**All studies**						
OS	27	4899	1.54 (1.32, 1.80)	<0.00001	63	<0.00001
DFS	5	1515	1.83 (1.31, 2.55)	0.0004	75	0.003
**Cancer types**						
Hepatocellular Carcinoma	3	410	1.50 (1.17, 1.93)	0.001	0	0.81
Colorectal cancer	2	1065	2.03 (0.70, 5.84)	0.19	63	0.1
Esophageal squamous cell carcinoma	3	471	1.89 (1.42, 2.52)	<0.0001	0	0.66
Gastric cancer	3	411	1.66 (0.88, 3.14)	0.12	90	<0.0001
Breast cancer	10	1645	1.31 (1.10, 1.55)	0.002	0	0.98
Other cancer types	6	897	1.72 (1.07, 2.79)	0.03	86	<0.00001
**HR estimation**						
Multivariate analysis	16	2917	1.69 (1.26, 2.27)	0.0005	80	<0.00001
Univariate analysis	11	2812	1.90 (1.48, 2.42)	<0.00001	66	0.001
**Method**						
PCR	13	1633	1.46 (1.25, 1.70)	<0.00001	0	0.79
IHC	14	3266	1.67 (1.31, 2.14)	<0.0001	79	<0.00001
**Cut off value**						
≥median	11	1911	1.29 (1.05, 1.60)	0.02	49	0.02
scores of 4 (≥26% of tumor cells)	11	2768	1.81 (1.43, 2.30)	<0.00001	68	0.0006
other	2	220	1.80 (1.12, 2.90)	0.01	66	0.08
**Ethnicity**						
Asian	17	2620	1.69 (1.45, 1.98)	0.001	59	<0.00001
European	7	1971	1.01 (0.80, 1.29)	0.91	64	0.01
North American	3	308	1.30 (0.86, 1.98)	0.42	0	0.95

OS, overall survival; DFS, Disease-free Survival; IHC, Immunohistochemistry; HR, hazard ratio.

### Study Methodology and Study Quality Assessment

The technical detail and the study methodology for SIX1 protein quantification is also shown in **Table 1**. There were 14 studies that analyzed SIX1 mRNA expression using qPCR and 13 studies that quantified tumor protein expression by using IHC. We did not identify any study that quantified SIX1 protein expression level in tumor lysate. Most of the studies analyzed the expression of other factors with SIX1 simultaneously, found in 20 studies (**Table 1**).

The method for defining low and high SIX1 expression level was reported in 89% of studies, with 33% of studies defined high SIX1 expression as above median value, 40% of studies based on IHC scores (4, ≥26% positive cells), only one of the studies determining SIX1 value cutoffs based on ROC curve analysis.

### Survival Analysis

**Figure 2:**
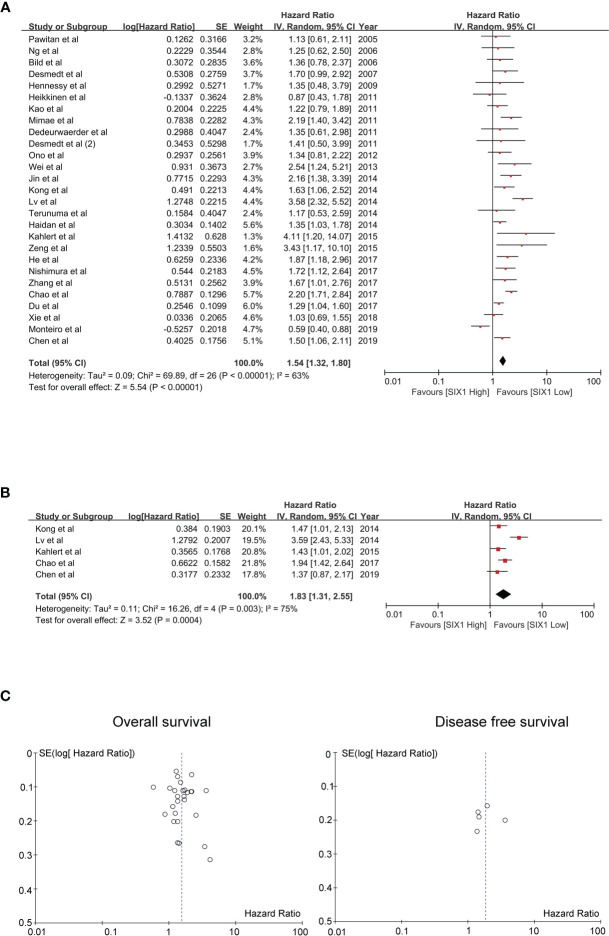
Evaluating HRs of SIX1 expression and the prognosis of cancer patients. **(A)** Forest plot of studies evaluating high expressed SIX1 and the OS; **(B)** Forest plot of studies evaluating high expressed SIX1 and the DFS; **(C)** Funnel plots of studies evaluating OS (left) and DFS (right).

The pooled HR for overall survival in patients with high SIX1 expression compared with low expression was 1.54 (95% CI: 1.32-1.80, *P*<0.00001), but with a significant degree of heterogeneity (*I^2 =^* 63%) (**Figure 2A**), while the pooled HR for disease-free survival was 1.83 (95% CI: 1.31-2.55, *P*=0.0004), again with a high degree of study heterogeneity (*I^2 =^* 75%) (**Figure 2B**). Funnel plots for overall, disease-free survival demonstrated no evidence of publication bias or small study effects (**Figure 2C**). The major cause of the heterogeneity was because of the relationship between SIX1 expression and the outcome of osteosarcoma.

### Subgroup Analysis

**Figure 3:**
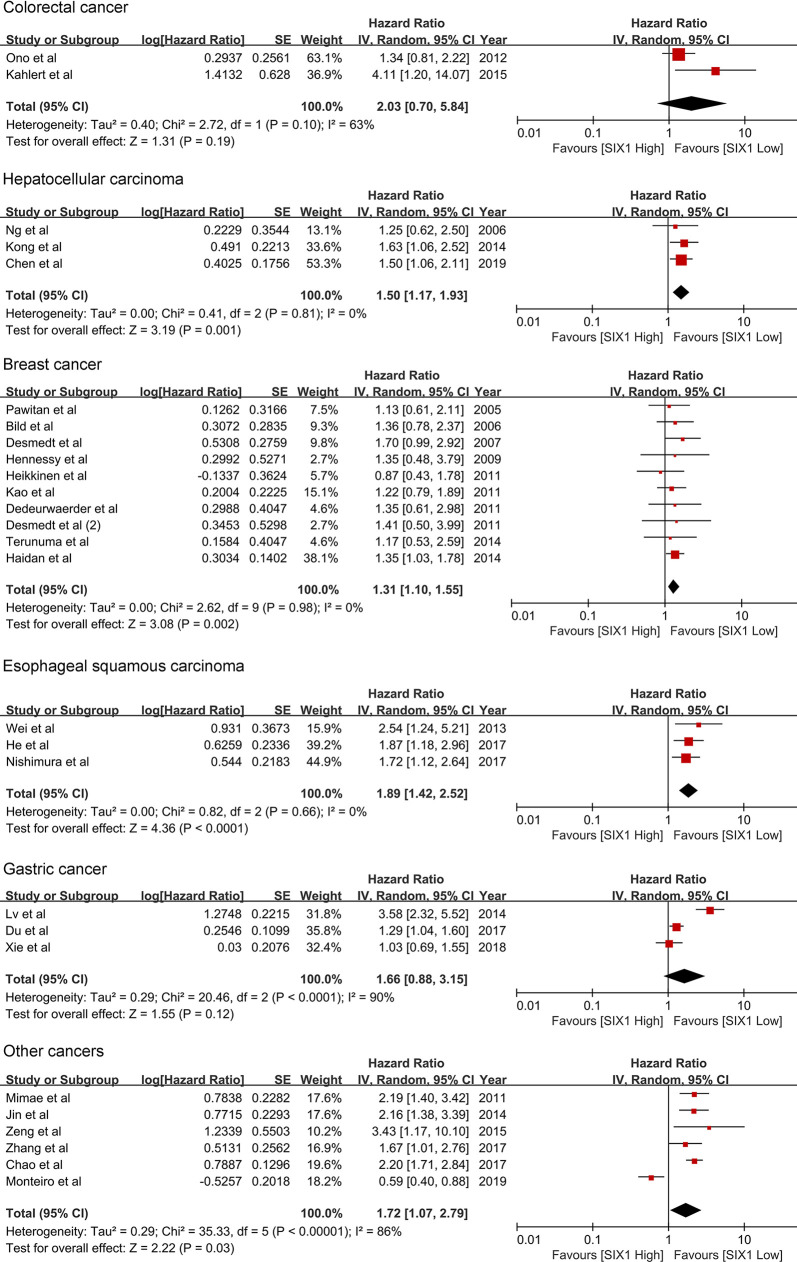
Forest plot of subgroup analysis by cancer type and OS.

The subgroup analysis showed that the SIX1 expression is associated with reduced overall survival in hepatocellular carcinoma (HR 1.50; 95% CI: 1.17-1.93, *P* =0.001), Esophageal squamous cell carcinoma (HR 1.89; 95% CI: 1.42-2.52, *P*<0.0001) and breast cancer (HR 1.31; 95% CI: 1.10-1.55, *P* =0.002). (**Table 2** and **Figure 3**) For colorectal and gastric cancer, however, there was no relationship between SIX1 expression and overall survival (HR 2.03; 95% CI: 0.70-5.84, *P* =0.19) and (HR 1.66; 95% CI: 0.88-3.15, *P* =0.12), respectively.

We further performed subgroup analyses based on defining SIX1 expression method, reasoning that this analysis might help differentiate the studies with high level of heterogeneity (**Table 2**).

### SIX1 Overexpression Level and Its Relative Clinical Parameters

To evaluate SIX1 precisely of its impact on clinical outcome, we investigated the correlations between SIX1 expression level and the clinical parameters with included cancers (**Table 3**). We found that the expression levels of SIX1 was related with the histological grade (HR=2.00, 95% CI=1.04, 3.86, *P*=0.04), tumor size (HR=2.11, 95% CI=1.22, 3.65, *P* =0.007), lymph node metastasis (HR=1.97, 95% CI=1.32, 2.93, *P* =0.0008), clinical stage (HR=3.34, 95% CI=1.86, 5.98, *P*<0.0001), TNM stage (HR = 3.87, 95% CI = 2.03, 7.39, *P*<0.0001),significantly. While there were no significant associations between SIX1 expression and gender, age, distant metastasis. These correlations indicated that SIX1 high expression was associated with the malignant biological behaviors in cancers (**Table 3**).

**Table 3:** Clinicopathological features and the high expressed SIX1 in patients with cancer.

Clinicopathological parameters	Number of studies	Number of patients	Model	Risk of high SIX1	Significant Z	*p*-value	Heterogeneity	
				OR 95%CI			*I^2^* (%)	*P_H_*
**Gender** (Male *vs.* Female)	14	2484	Fixed	0.99 (0.83, 1.18)	0.1	0.92	40	0.07
**Age**								
>25 *vs.* ≤25	1	100	—	0.90 (0.40-2.01)	—	0.80	—	—
>45 *vs.* ≤45	1	160	—	0.66 (0.25-1.74)	—	0.40	—	—
>50 *vs.* ≤50	6	1055	Fixed	0.97 (0.74, 1.27)	0.24	0.81	17.0	0.30
>55 *vs.* ≤55	1	103	—	1.168 (0.53-2.60)	—	0.70	—	—
>60 *vs.* ≤60	3	322	Fixed	1.09 (0.69, 1.73)	0.37	0.71	0	0.55
>65 *vs.* ≤65	1	144	—	1.09 (0.57-2.11)	—	0.79	—	—
**Histological grade** (poor *vs.* well and moderate)	9	2320	Random	2.00 (1.04, 3.86)	2.07	0.04	88	<0.00001
**Tumor size** (large *vs.* small)	9	1248	Random	2.11 (1.22, 3.65)	2.68	0.007	70	0.0007
**Venous infiltration** (present *vs.* absent)	2	265	Random	6.89 (3.82, 12.42)	6.41	0.02	83	<0.00001
**Lymph node metastasis** (positive *vs.* negative)	9	2155	Random	1.97 (1.32, 2.93)	3.34	0.0008	67	0.002
**Distant metastasis** (positive *vs.* negative)	4	562	Fixed	1.36 (0.83, 2.21)	1.23	0.22	0	0.85
**Clinical stage** (late *vs.* early)	10	2271	Random	3.34 (1.86, 5.98)	4.04	<0.0001	84	<0.00001
**TNMstage** (III, IV *vs.* I,II)	5	632	Random	3.87 (2.03, 7.39)	4.1	<0.0001	63	0.03
**Recurrence** (Yes *vs.* No)	7	1469	Fixed	1.39 (1.13, 1.72)	3.11	0.002	0	0.66
**Other effects** (positive *vs.* negative)								
AFP (20ng/ml)	2	265	Random	1.13 (0.52, 2.46)	0.3	0.76	55	0.14
HBsAg status	2	264	Random	0.83 (0.27, 2.55)	0.33	0.74	61	0.11
ER	5	698	Fixed	0.88 (0.64, 1.21)	0.79	0.43	33	0.2
PR	2	351	Fixed	1.26 (0.80, 1.99)	0.98	0.33	4	0.31
Basal/Luminal	5	724	Fixed	0.44 (0.29, 0.68)	3.75	0.0002	0	1

OR, Odds ratio; AFP, alpha fetoprotein; ER, Estrogen receptors; PR, Progesterone receptors.

### Sensitivity Analysis and the Publication Bias Evaluation

**Figure 4:**
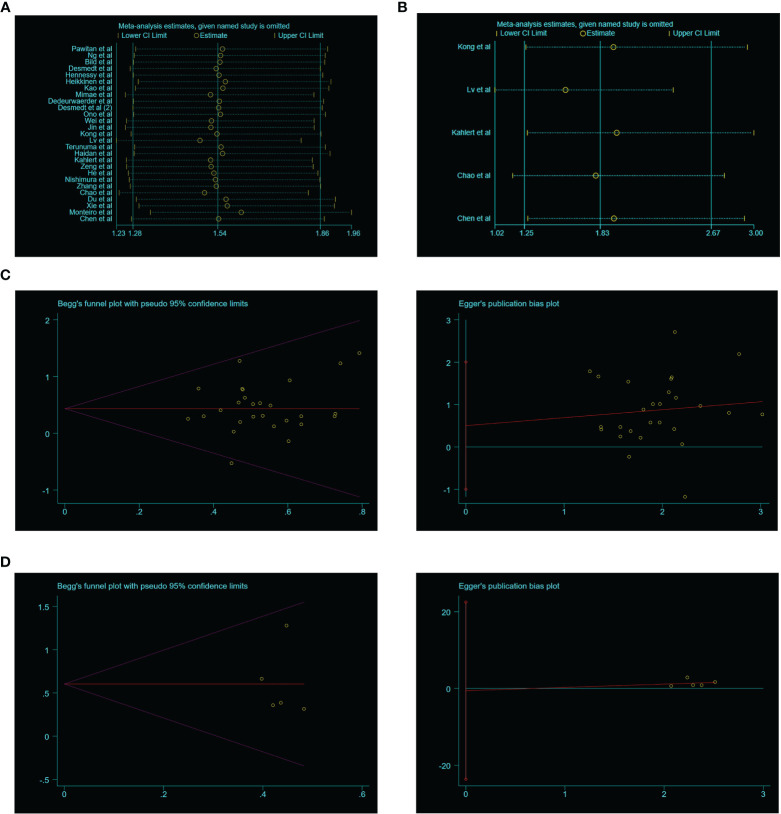
Sensitivity analysis and publication bias of the meta-analysis. **(A)**. OS of SIX1 expression levels; **(B)**. DFS of SIX1 expression levels; **(C)**. OS of Begg’s funnel plots(left) and Egger’s plots (right); **(D)**. DFS of Begg’s funnel plots(left) and egger’s plots (right).

We performed sensitivity analysis to analyze whether a single study could affect the overall result. Results showed that the associated studies between SIX1 expression and OS and DFS demonstrated that the single study cannot influence the result of meta-analysis (**Figures 4A, B**). Begg’s test and Egger’s test were used, and showed there is no publication bias existed in the studies on associations between SIX1 overexpression level and OS (*P* = 0.497), DFS (*P* = 0.940) (**Figures 4C, D**).

### SIX1 Expression Data and the TCGA Online Datasets

**Figure 5:**
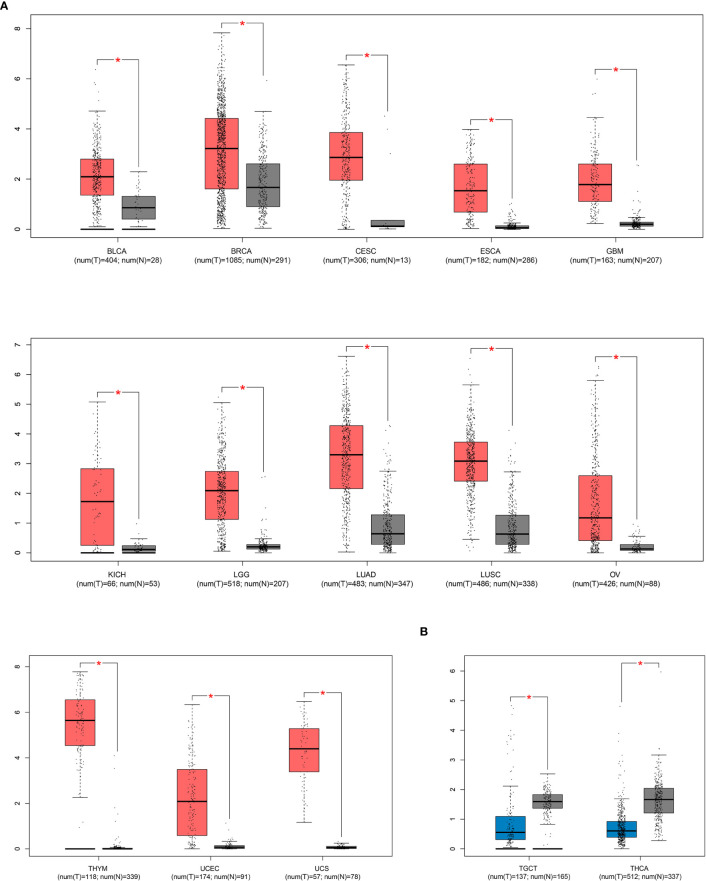
TCGA data sets represent SIX1 aberrant expression levels in various cancer types. **(A)**. SIX1 overexpressed cancer types; **(B)**. SIX1 lower expressed cancer types. * means P value < 0.05 have statistical significance.

The SIX1 expression level in various tumors were performed by GEPIA, a public online database that provide customized survey based on TCGA and GTEx datasets. SIX1 expression is detected in 13 types of cancers and demonstrated that SIX1 expression is significantly higher in cancer tissues than in the normal tissues (**Figure 5A**). Besides, we found in Testicular germ cell tumor (TGCT) and Thyroid carcinoma (THCA) the SIX1 expression is inversely downregulated, which are not included in our meta-analysis (**Figure 5B**).

### Validation of Prognostic Correlations in TCGA Online Datasets

**Figure 6:**
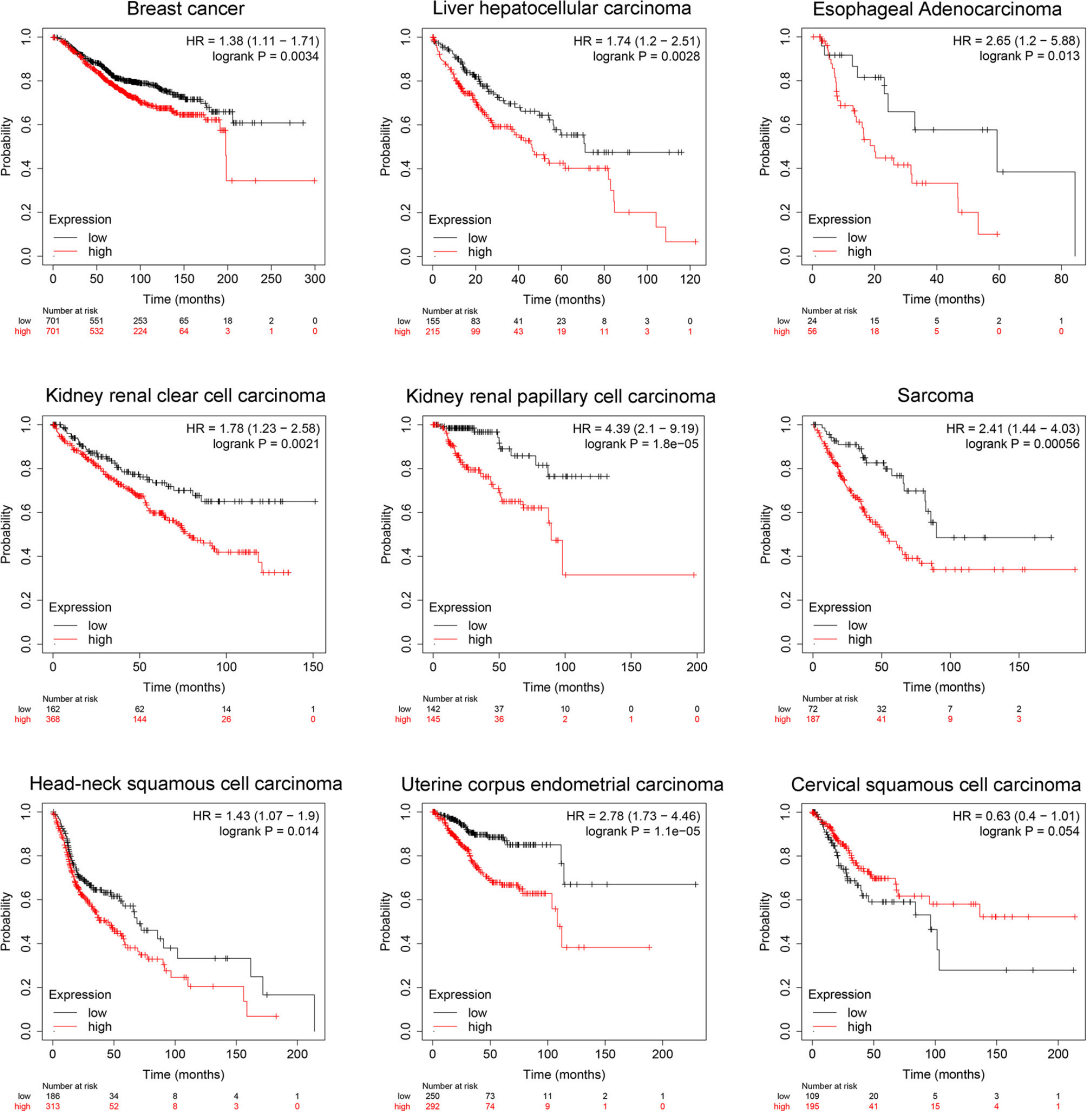
TCGA data sets represent SIX1 aberrant expression levels predicts poor overall survival in various cancer patients.

To validate the clinical prognosis value of SIX1, we explored TCGA datasets by KM-plotter, an interactive online tool that analyze the expression data of genes based on TCGA. The significant association between high SIX1expression and poor OS was found in 9 types of cancers (**Figure 6**). The result of our meta-analysis and TCGA datasets validation demonstrated the correlation between the SIX1 expression level and the breast cancer, liver hepatocellular carcinoma. The OS data of the other seven cancer types were provided for additional information.

### PPI Network and Functional Enrichment Analysis

To further analyze the molecular mechanisms of SIX1, we generate PPI networks of SIX1-related genes obtained by STRING, totally observed 10 nodes and 23 edges (**Supplementary Figure 1A**). SIX1-related genes were collected for biological functional enrichment analysis (**Supplementary Figure 1B**). The top significant terms, contained the biological processes, cell components and molecular function, and the most significant were selected. These SIX1-related genes were significantly enriched in DNA binding, catalytic activity, and transcription binding.

## Discussion

Recently, increased evidence indicated that SIX1 is involved in multiple process of tumor development and indicated the expression level of SIX1 could be a biomarker for assessing the prognosis in tumors. However, limited by insufficient data and complicated experimental model, single study usually meets the restrictions easily, and the data is unreliable. Therefore, a meta-analysis based on pooled studies is necessary for discovering the potential clinical value of SIX1 expression and the correlation in cancer patient prognosis.

The primary aim of this meta-analysis is to determine whether SIX1 is associated with survival outcome in cancer patients. We discovered that high SIX1 expression is associated with poorer overall survival in patients with breast cancer, hepatocellular carcinoma, and Esophageal squamous cell carcinoma. Our data indicated that SIX1 levels is helpful for predicting patient’s prognosis in these cancer types. Although some studies invalid SIX expression analysis studies were excluded from our study pool, some published studies that assessed SIX1 mRNA level based on TCGA datasets supported our findings, indicating that SIX1 aberrant expression and poor patient prognosis in sarcoma, but we failed to find relative articles for further analyze.

Our second purpose is to discover whether SIX1 can be used as a prognostic biomarker in cancer patients. This requires large quantity of clinical samples and be carefully analyzed. Most of the studies included were of a prospective and retrospective, some were purely retrospective. These may result in a risk of bias. The bias is reassured by the funnel plots, extracted data from studies that reporting overall and disease-free survival, also demonstrated no evidence of publication bias. Both results were supported by both Begg’s funnel plots and Egger’s plots. This suggests that the nonsignificant findings associated between SIX1, and outcome are somehow published frequently and lower the inaccuracy of our analysis.

For being a helpful prognostic biomarker, SIX1 must display clinical utility, analytic and clinical validity. For studies that meet the included criteria, the SIX1 potential clinical validity is examined in the ‘subgroups’ analysis. Clinicopathological features analysis displayed that the SIX1 overexpression was linked with poor histological grade, tumor size, venous infiltration, lymph node metastasis, late clinical stages and advanced TNM stages. These data indicated that there is a correlation between SIX1 expression and advanced cancer features. Subgroup analysis based on cancer types showed the associations between overexpressed SIX1 and poor overall OS in cancer patients. SIX1 is significative in the hepatocellular carcinoma, Esophageal squamous cell carcinoma, and breast cancer, but not in the colorectal cancer and gastric cancer. This was also verified by TCGA online database and displayed in KM curves in **Figure 6**.

We next evaluated the SIX1 expression and its impact on clinical outcome different clinical parameters were included. Expression levels of SIX1 was related with the histological grade, tumor size, lymph node metastasis, clinical stage, TNM stage, significantly. These data indicated SIX1 is associated with tumorigenesis and tumor growth, accelerates tumor progression. This may also give images on SIX1 is essential in cancer cell proliferation, for SIX1, in another point of view, plays key roles in embryonic development and cell grown. Though there were no significant associations between SIX1 expression gender, age, and distant metastasis, we found that SIX1 has its roles in lymph node metastasis, clinical stage and TNM stage. We believe that SIX1 is an important regulator in cancer cell metastasis, but the result showed that SIX1 is unrelated with distant metastasis may be due to lacing of clinical samples and the refractory of late staged cancer patients carrying distant metastasis.

Although, SIX1 was found highly expressed in various malignancies and was related to a more aggressive phenotype, late clinical stage, and poor prognosis. The exact mechanisms of how SIX1 promotes tumorigenesis remains unclear (2). The gene functional enrichment analysis showed that SIX1 is a critical transcription factor in tumorigenesis, SIX1 related genes significantly enriched in DNA, E-box binding and DNA transcription regulation. Reports shown that SIX1 overexpression activate breast cancer cell proliferation and tumorigenesis by activating cyclin A1 transcription directly (47). In 2006, Ng et al. demonstrated that SIX1 is significantly associated with the recurrence and metastasis of HCC and SIX1 mRNA overexpression was observed in 85% of the HCC (21). This molecule is also involved in the Epithelial-to-mesenchymal transition (EMT) and was described as its activation of ZEB1 transcription and TGF-β signaling regulation, both found in colorectal and cervical cancer (48, 49).

The data displayed in this study only provided limited information about the clinical utility of SIX1 and were in specific groups of cancer patients. This might because of the failure of some of the included studies did not provide adequately the numbers of the cancer patients. We found that even some basic data such as age, sex, clinical features like tumor size, tumor stage was not always recorded. Future studies therefore better determine and report the analysis clearly in a subset of cancer patients with the basis of histopathological and clinical utility of SIX1 expression (50, 51). Hence, addition of the online datasets supplement provides a more convinced display.

In summary, this meta-analysis used multiple search strategies identified a wide range of studies from different populations, clinical parameters-based subgroup analysis and highlighted the potential differences in each relationship associated with SIX1 expression and the prognosis in various cancer types. This systematical assessment, we believe will perform a guidance, and it will ensure the improvement of higher quality treatment data in the SIX1 investigations as a prognostic biomarker. There also require balances of the facts that some included criteria failed to blind the assessor on SIX1 and the outcome status evaluation may lead to a risk of reporter bias. Overall, the quality of this study still needs improvement, and more progress is needed to make a better defining of SIX1 expression and its roles in cancers.

## Data Availability Statement

The original contributions presented in the study are included in the article/**Supplementary Material**. Further inquiries can be directed to the corresponding author.

## Author Contributions

YP and ZL helped analyze the data. GZ drafted and write the paper. YL and LZ revised the paper. All authors contributed to the article and approved the submitted version.

## Funding

This work was supported by the Funds for Local Science and Technology Development Guided by the Central Committee, basic research project of Jilin Province (202002021JC) and National Natural Science Foundation of China (No. 81860651).

## Conflict of Interest

The authors declare that the research was conducted in the absence of any commercial or financial relationships that could be construed as a potential conflict of interest.

## Publisher’s Note

All claims expressed in this article are solely those of the authors and do not necessarily represent those of their affiliated organizations, or those of the publisher, the editors and the reviewers. Any product that may be evaluated in this article, or claim that may be made by its manufacturer, is not guaranteed or endorsed by the publisher.

## Supplementary Material

Supplementary Figure 1 Mechanisms prediction of SIX1-related genes with bioinformatics and enrichment. **(A)** The protein-protein interaction network of SIX1-related genes. **(B)** The functional enrichment analysis of SIX1-related genes.
