# Supplementary figures and images for: The Significance of SIX1 as a Prognostic Biomarker for Survival Outcome in Various Cancer Patients: A Systematic Review and Meta-Analysis
Source: Front Oncol. 2021 Oct 21;11:622331. doi: 10.3389/fonc.2021.622331 (PMC8567106; doi:10.3389/fonc.2021.622331)

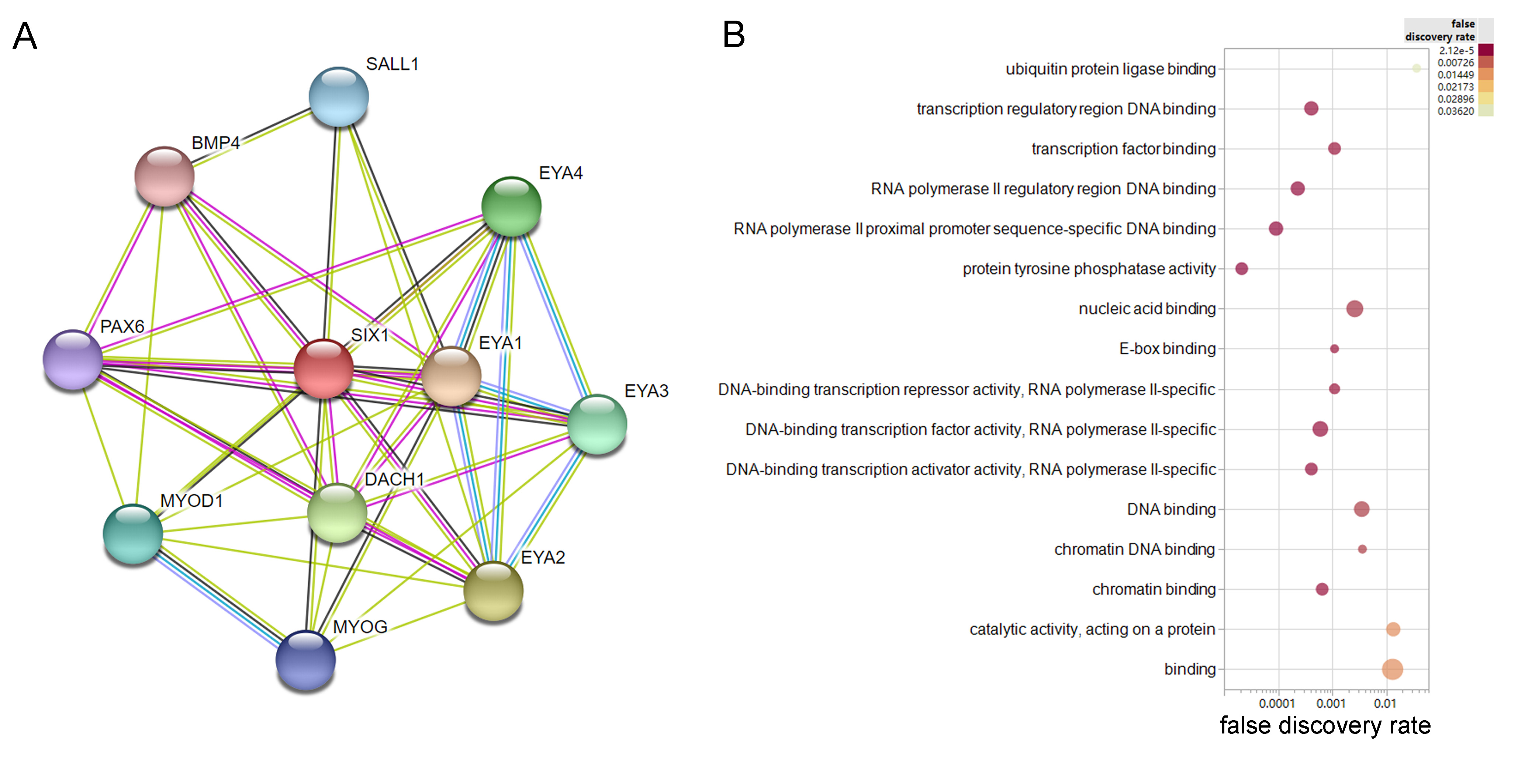

Supplement: Supplementary Figure 1 — Mechanisms prediction of SIX1-related genes with bioinformatics and enrichment. (A) The protein-protein interaction network of SIX1-related genes. (B) The functional enrichment analysis of SIX1-related genes. [file Image_1.jpeg]
